# Preferences of public sector medical doctors, professional nurses and rehabilitation therapists for multiple job holding regulation: A discrete choice experiment

**DOI:** 10.1371/journal.pone.0320854

**Published:** 2025-04-15

**Authors:** Busisiwe Precious Matiwane, Laetitia C. Rispel, Duane Blaauw

**Affiliations:** 1 Centre for Health Policy & South African Research Chairs Initiative (SARChI), School of Public Health, University of the Witwatersrand, Johannesburg, South Africa; 2 Centre for Health Policy, School of Public Health, University of the Witwatersrand, Johannesburg, South Africa; University of Salerno - Baronissi, ITALY

## Abstract

**Introduction:**

Regulating multiple job holding (MJH) among health professionals is challenging for many health systems. The effectiveness of different MJH policy reforms depends on the behavioural responses of different groups of health professionals but little is known about their preferences and likely reactions.

**Aim:**

Investigate the preferences of public sector medical doctors, professional nurses, and rehabilitation therapists for different MJH regulations in two South African provinces.

**Materials and methods:**

We developed a novel discrete choice experiment (DCE) to evaluate the preferences of health professionals for jobs with varying MJH policy interventions. The DCE attributes included *restrictive* regulations (banning MJH) versus *reward-oriented* policies (increased public sector salaries, expanded overtime, improved clinical practice environment, and better hospital management). We produced an unlabelled DCE using an efficient design and administered it to a representative sample of health professionals. Generalized multinomial logit models were used for analysis. We also investigated group heterogeneity, calculated marginal willingness to pay and estimated uptake for different policies.

**Results:**

1387 participants completed the DCE. The doctors, nurses and rehabilitation therapists were strongly opposed to banning MJH, requiring salary increases of 45.7%, 20.0% and 42.8%, respectively, to accept an MJH ban. Increased public sector salaries significantly increased public sector retention. However, non-financial interventions were also influential. Doctors, nurses, and rehabilitation therapists were willing to forgo 57.9%, 54.8%, and 38.9% of their salaries, respectively, for an improved clinical practice environment. Competent hospital management was also important. There was some preference heterogeneity. Nurses had significantly different preferences for certain attributes compared to the other two groups, and professionals currently engaged in MJH were significantly more opposed to banning MJH.

**Conclusion:**

This study provides new information on health professional preferences for different MJH regulations. It confirms the importance of non-financial policy interventions in addressing MJH and the need to tailor MJH policy design.

## Introduction

Multiple job holding (MJH) or dual practice, where individuals engage in more than one paid job simultaneously, has received increasing attention from labour market analysts, policymakers and researchers [1–4]. Variations in MJH across different labour markets are driven by differences in job opportunities, pay differentials, regulatory regimes and individual worker preferences [5,6].

Many public sector health professionals have a second job in the private sector [5]. This phenomenon has been reported in a range of different countries, at least for doctors [6,7] and nurses [8]. Theoretically, MJH by public health professionals could have contradictory effects on the performance of public healthcare systems [7,9]. On the one hand, MJH might increase the retention of skilled health professionals in the public sector, at a lower cost, which would enhance access, quality, efficiency and equity [10–13]. On the other hand, MJH professionals may decrease time and effort in their public jobs, suffer fatigue and burnout, divert profitable patients to their private practices, or appropriate public resources for private use, which would all compromise public system access, quality, efficiency and equity [6,14,15]. However, there is very little empirical evidence on the balance of those potential impacts in different settings, nor on the contextual factors that may promote more positive outcomes [16,17].

Different countries have adopted diverse strategies to regulate MJH among health professionals [17–19]. A few countries rely mainly on professional bodies and peers to regulate unprofessional and unethical practice [9]. *Restrictive* MJH policies include banning MJH completely, limiting it to specific groups of professionals, specifying maximum hours of permitted MJH, or restricting private practice earnings from MJH [18]. Other countries have focused on *reward-oriented* MJH policies which include raising public sector salaries, using non-financial incentives to encourage retention, offering more lucrative contracts to health professionals who agree to work exclusively in the public sector, or encouraging the development of private practices within public facilities [9,18].

Some researchers have developed theoretical economic models to explore the dynamics of MJH and predict the likely impact of different policy interventions [12,20–23]. There are also some narrative descriptions of individual country experiences with MJH regulation [18,24]. However, there are few rigorous empirical evaluations of the impact of MJH policies in the health sector, probably due to the methodological difficulties of designing such studies [17]. Indeed, the Cochrane systematic review of interventions to manage the MJH of health professionals found not a single eligible study to include in the review [25].

The likely impact of different MJH policies is determined by the behavioural responses of health professionals to policy changes. Surprisingly, this is an underexplored area in the health MJH literature [26], even though the methodology is less of an obstacle. The model of Gonzalez and Macho-Stadler [21] makes theoretical assumptions about the probable motivations and responses of health professionals to MJH regulation but empirical studies are rare. For example, Jumpa *et al*’s [27] qualitative interviews with 20 Peruvian doctors included some discussion about their attitudes to MJH regulation. Our study attempts to address this knowledge gap by using a discrete choice experiment (DCE) to investigate the preferences of South African health professionals for different MJH regulatory policies.

The South African healthcare system continues to grapple with poor health outcomes, persistent health inequalities, and weak public sector management [28,29]. South Africa (SA) has a mixed healthcare system with a well-developed private sector. The private sector accounts for approximately 50% of national health expenditure, and employs over 60% of health professionals, even though it only services the most affluent 17% of the population able to afford private health insurance [30]. In SA, the Public Service Amendment Act and regulations make legal provisions for MJH, known as remunerative work outside of the public sector (RWOPS) [31–33]. The RWOPS policy was introduced to encourage the retention of health professionals in the public sector. It allows public sector employees to engage in additional paid work in the private sector provided that they have written permission from the relevant authority and that such work is performed outside of their public sector working hours [34]. There has been no formal evaluation of the impact of the RWOPS policy although one government investigation found evidence of abuse by doctors and weak monitoring [35]. There is also limited academic literature on RWOPS in SA [36].

DCEs are a quantitative methodology for evaluating the relative importance of different product characteristics on consumer choices [37]. In health, DCEs have mainly been used to assess patient preferences for different models of healthcare delivery [38,39] or health workers’ valuation of different job characteristics affecting their job choices [40–42]. This paper adds to the small number of studies that have used DCEs to investigate aspects of MJH. Scott *et al* [43], included a baseline job characteristics DCE in their panel study with Australian medical specialists. One of the job attributes was the percentage of time spent in the private sector, which they then used to analyse the general preference for private sector work and its association with risk aversion. More recently, Pestana *et al* [44] did a standard job characteristics DCE with doctors in Portugal but compared the preferences of doctors working exclusively in the public sector with those engaged in MJH.

Our application is more novel. We developed a new DCE to investigate the preferences of South African public health professionals for different MJH policy interventions. We evaluate the trade-offs between *restrictive* regulations such as banning MJH, and *reward-oriented* policies such as an increase in public sector salaries or introducing other non-financial incentives to improve public sector retention. The study was done with public sector medical doctors, professional nurses, and rehabilitation therapists from two SA provinces. The inclusion of rehabilitation therapists and the comparison between the three professional groups are also novel in the literature. Information about the preferences and choices of health professionals will aid in predicting their likely behavioural responses to policy interventions which can inform the design and implementation of more effective MJH regulation.

## Materials and methods

### Study participants

The study was conducted from July 2022 to October 2022 at 29 public hospitals in two South African provinces, Gauteng (GP) and Mpumalanga (MP). The DCE was part of a cross-sectional survey on MJH conducted with medical doctors (generalists and medical specialists), professional nurses, and rehabilitation therapists (occupational therapists, physiotherapists, speech therapists and/or audiologists) in the study hospitals in 2022. The initial calculated sample comprised 552 doctors and 502 nurses, evenly distributed between the two provinces, along with 237 rehabilitation therapists in Gauteng and 152 rehabilitation therapists in Mpumalanga.

### Sampling methodology

We recruited public sector doctors, nurses, and rehabilitation therapists from 56 hospitals in Gauteng and Mpumalanga using a multi-stage sampling approach. A stratified sample of 11 hospitals per province was selected randomly from four categories (strata) of hospitals (district, regional, tertiary and central) through a proportional-to-size strategy [45]. GP’s sample included five district, two regional, two tertiary, and two central hospitals, while MP’s sample included six district, three regional, and two tertiary hospitals, as it has no central hospitals [46].

Within selected hospitals, six clinical disciplines anaesthesia, orthopaedics, paediatrics, obstetrics and gynaecology, internal medicine, and surgery were targeted. Wards within these disciplines were randomly chosen, and all doctors and nurses in the selected wards were invited to participate until target numbers were met. Rehabilitation therapists were recruited from 22 hospitals due to their smaller population size. Additional hospitals, four in MP and three in GP were included to meet sample size requirements for rehabilitation therapists [46].

### DCE design

We followed the recommended standards for conducting a DCE study [47–49]. We used an unlabelled design with two alternatives plus an opt-out in each choice set. Respondents were asked to select between two public sector jobs (Job 1 and Job 2) containing various combinations of incentives and restrictions of MJH practice. The opt-out allowed respondents to refuse both of the choices offered to better reflect real-world labour market choices [49,50].

Several steps were involved in selecting the attributes and levels to be included in the design experiment. First, a literature review on MJH and MJH regulation was undertaken. In-depth interviews were then conducted with key stakeholders across South Africa to gather their perspectives on the effectiveness of current MJH policies and identify potential regulatory mechanisms or enhancements to address MJH. In-depth interviews were also conducted with doctors, nurses and rehabilitation therapists who have engaged in MJH, focusing on their motivations for participating in MJH, their views on the current regulatory mechanisms for MJH, and possible new incentives and restrictions related to MJH. Based on the qualitative findings, we designed a DCE to evaluate the preferences of health professionals for different MJH regulations versus different financial and non-financial incentives intended to make public sector jobs more attractive and thereby decrease MJH.

The final list of attributes and levels is shown in **Table 1**. Current RWOPS regulations in SA allow MJH after normal hours with restrictions. The DCE evaluated more restrictive (banning MJH) and more permissive (extended MJH hours and MJH permitted during normal hours) regulatory regimes. Salary supplementation is the main reason in the literature for MJH participation among health professionals [11,14–16], and a salary increase would be the main financial incentive to compensate health professionals for accepting MJH restrictions. This was included as a percentage change in current salary levels to allow comparison between the different professional groups, with four levels to be able to evaluate nonlinear effects. This attribute was also used to calculate willingness-to-pay estimates in terms of salary compensation for the other attributes. Four different employment contracts were included in the DCE design because they have financial impacts that may influence MJH and because more flexible arrangements could be useful in the management of MJH. A part-time post would allow more time in the second job and might better reflect the hours currently worked by professionals engaging in MJH. Increased overtime in the primary job would be an alternative form of income supplementation to MJH. Two non-financial interventions that may influence MJH were also included, with two levels each. The clinical practice environment with adequate resources and staffing to enable quality care has been shown to be important in job selection between private and public sectors [11]. The final attribute focused on improved hospital management, particularly in relation to the management of MJH (RWOPS).

**Table 1:** DCE attributes and levels.

Attribute	Attribute levels
Employment contract	Part-time (5/8) post with no paid overtime Full-time post with no paid overtime Full-time post with 8 hours/week paid overtime Full-time post with 16 hours/week paid overtime
Current salary	No salary increase 25% salary increase 50% salary increase 75% salary increase
Clinical practice environment	Shortages of staff, drugs, equipment, and infrastructure results in the provision of sub-optimal health care to patients Availability of staff, drugs, equipment, and infrastructure results in the provision of good quality health care to patients
Conditions for RWOPS (MJH)	RWOPS (MJH) prohibited Approved RWOPS allowed, but only after working hours and limited to 8 hours per week Approved RWOPS allowed, but only after working hours and limited to 16 hours per week Approved RWOPS allowed during working hours but limited to 8 hours per week
Hospital management	Sub-optimal management, unable to manage RWOPS in the hospital Competent management, able to manage RWOPS in the hospital

The final DCE design yielded 256 (4^3^ x 2^2^) potential combinations. We first used dcreate in Stata [38] to generate an orthogonal experimental design with zero priors. This was used in the pilot study in two unsampled public hospitals to further refine the attributes and levels and to obtain priors to be used in producing the final design [50]. The final experimental design was generated using an efficient design strategy in Ngene with 16 choice sets. To minimise task fatigue the design was split into two blocks [51]. We also repeated one choice set to assess internal consistency and verify that participants actively participated in the task [51]. Thus, each participant answered nine choice sets in the survey. Each block was presented in two versions, with the order of choice tasks reversed in the second version to account for ordering effects. The four versions of the tool were assigned to participants randomly.

### Data collection

**Fig 1:**
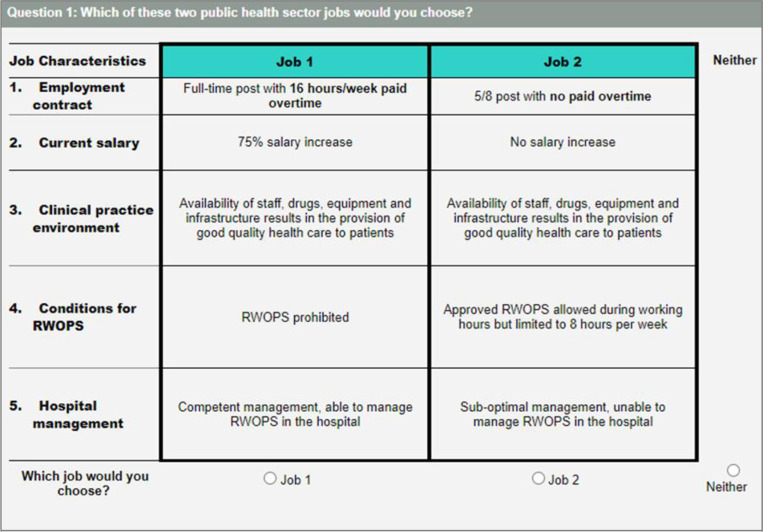
DCE question example.

Consenting study participants completed the self-administered questionnaire anonymously on tablets using REDCap (Research Electronic Data Capture) [52]. The DCE tool contained an introductory section explaining the task, attributes and levels (Supplemental File 1), and a practice question. An example of the DCE choice task presented to respondents is shown in **Fig 1**. The questionnaire also included questions on socio-demographics and engagement in MJH during the preceding 12 months.

### Data analysis

Data was analysed in Stata 17. We used Stata’s *svy* commands to account for the complex cluster sampling design, as explained previously [46]. Separate DCE analyses were conducted for the three professional groups. The results were weighted to reflect the population distribution of the study health professionals for different hospital types within the two provinces. Ten participants who did not make trade-offs were excluded from the DCE analysis. We compared the conditional logit model (clogit), mixed logit (MIXL) and generalised multinomial logit models (G-MNL). For the MIXL and G-MNL-II models, we used the Stata *mixlogit* [53] and *gmnl* [54] ado commands, respectively with appropriate weighting. The G-MNL model had the best fit. The G-MNL model accommodates both preference and scale heterogeneity [55]. Accounting for scale heterogeneity is important in comparing groups which may differ in the variance of their error terms [55]. The DCE results presented here are from the uncorrelated G-MNL-II model, using the responses which included the opt-out, with all parameters as random estimated with 500 Halton draws, and gamma equal to zero. We use the mean regression coefficients and confidence intervals to determine the relative importance of attributes, while the standard deviations of the coefficients in the G-MNL models reflect preference heterogeneity within the sample. Categorical attributes were entered as dummy variables for each level. We compared the categorical and numerical treatment of the salary increase attribute. The results presented are for the numerical analysis of a linear 10% salary increment in all models.

Because individual characteristics do not vary within choice sets, their impact on preferences is evaluated statistically by including interaction terms between individual and design variables in the models [37]. Three different interaction models were used to assess the influence of health professional group, MJH experience and socio-demographic factors on attribute preferences. The socio-demographic characteristics evaluated were gender, marriage status, having dependants, coming from an urban province and having a professional specialisation, which were shown to be influential in our previous analyses [46]. We did not include all possible interactions but developed more parsimonious final models that include effects of the most statistical significance or policy importance. Although preliminary latent class analysis showed consistent findings, it offered no additional insights on the outcomes of interest, so we prioritised the G-MNL model for its parsimony and relevance.

We estimated the marginal willingness to pay (mWTP) for each attribute in WTP space from the G-MNL model results. We did this using the Stata *gmnl* command [54], with the mean of the salary variable constrained to one, a fixed parameter for the opt-out alternative specific constant, and random coefficients for all other variables. The mWTP indicates the percentage of salary participants were prepared to forgo for improvements in other attributes, or the additional salary required to compensate them for attributes that decreased utility. Finally, to aid in the interpretation of the policy relevance of the regression results, we modelled the uptake of different hypothetical jobs with different combinations of attribute levels. The likelihood of choosing different scenarios was predicted using the estimates obtained from the G-MNL model [54]. Alternative scenarios were compared to the current status quo as a baseline, and results are expressed as the change in uptake from the baseline [53].

### Ethics

The Human Research Ethics Committee (Medical) of the University of the Witwatersrand in Johannesburg provided ethical approval for this study [M210262]. Study permission was also obtained from the relevant authorities in both provinces. All participants provided electronic informed consent by selecting ‘yes’ before completing the survey.

## Results

### Demographic characteristics

The survey response rate was 83.9%. **Table 2** displays the weighted demographic characteristics of the 1387 participants, which comprised 484 doctors (34.9%), 565 nurses (40.7%), and 338 rehabilitation therapists (24.4%). Rehabilitation therapists were the youngest group on average (32.4±8.7 years) while nurses were the oldest (43.7±10.4), with the doctors in between (39.9±9.7 years). Nurses (91.3%) and rehabilitation therapists (81.7%) were mostly female, but women made up only 44.5% of the doctors. Among doctors, 44.3% were specialists, compared to 32.1% of nurses. Rehabilitation therapists had the highest reported MJH participation at 38.9% (95% CI: 31.1% - 47.3%) in the previous 12 months, followed by doctors at 33.8% (95% CI: 26.4% - 42.1%) and nurses at 8.9% (95% CI: 6.8% - 11.6%). The differences in the prevalence of MJH among the three professional categories were statistically significant (X^2^ = 133.4, p<0.001).

**Table 2:** Study participant characteristics.

Characteristics	Doctors n (%)/ mean ±SD	Nurses n (%)/ mean ±SD	Rehabilitation therapists n (%)/ mean ±SD
n	484	565	338
Province			
Gauteng	395 (81.6%)	443 (78.4%)	241 (71.2%)
Mpumalanga	89 (18.4%)	122 (21.6%)	97 (28.8%)
Age	39.9 ± 9.7	43.7 ± 10.4	32.4 ± 8.7
Gender			
Male	264 (54.7%)	46 (8.2%)	56 (16.5%)
Female	217 (44.5%)	516 (91.3%)	276 (81.7%)
Other	4 (0.8%)	3 (0.5%)	6 (1.8%)
Marital status			
Single	153 (31.6%)	257 (45.5%)	200 (59.2%)
Married/Living together	301 (62.2%)	230 (40.7%)	129 (38.1%)
Divorced/Separated	22 (4.6%)	41 (7.3%)	5 (1.5%)
Widowed	8 (1.6%)	37 (6.5%)	4 (1.2%)
No. of dependents	4.2 ± 3.8	5.1 ± 3.4	2.4 ± 2.7
Category			
Generalist	269 (55.7%)	383 (67.9%)	338 (100.0%)
Specialist	215 (44.3%)	182 (32.1%)	NA
MJH (previous 12 months)			
No	320 (66.2%)	515 (91.1%)	207 (61.1%)
Yes	164 (33.8%)	50 (8.9%)	131 (38.9%)

MJH: multiple job holding, NA: Not applicable

### DCE results by professional group

#### G-MNL Regression.

**Table 3** shows the G-MNL DCE results with separate models for each professional group (models 3.1, 3.2, and 3.3) as well as a pooled model (model 3.4) including interaction terms to test for statistically significant differences in group preferences. The G-MNL-II model had a better model fit than the MIXL and clogit models (Supplemental File 2). The signs of statistically significant attributes were in the expected direction. The coefficients for the opt-out alternative-specific constants were negative and significant for doctors and rehabilitation therapists signifying a preference for the offered jobs. The standard deviations in the G-MNL models were statistically significant for many attributes indicating significant preference heterogeneity even within the professional groups, and the significant *tau* for most models confirmed the existence of some scale heterogeneity.

**Table 3:** DCE G-MNL results for the three health professional groups.

	*Model 3.1*				*Model 3.2*				*Model 3.3*				*Model 3.4*			
	Doctors				Nurses				Rehabilitation therapists				Pooled + Interactions			
	Mean (SE)		SD (SE)		Mean (S)		SD (SE)		Mean (SE)		SD (SE)		Mean (SE)		SD (SE)	
Full-time post, no paid overtime ^a^	—		—		—		—		—		—		—		—	
Part-time post, no paid overtime	-0.20 (0.14)		0.47 (0.38)		-1.30 (0.33)	***	-1.11 (0.57)		-0.32 (0.15)	*	0.80 (0.28)	**	-0.83 (0.14)	***	0.52 (0.15)	***
Full-time post, 8hrs paid overtime	0.63 (0.13)	***	0.17 (0.25)		0.08 (0.26)		-0.36 (0.51)		0.40 (0.13)	**	-0.27 (0.32)		-0.09 (0.11)		0.18 (0.10)	
Full-time post, 16hrs paid overtime	0.78 (0.17)	***	0.90 (0.19)	***	-0.19 (0.25)		-1.36 (0.49)	**	0.43 (0.15)	**	-0.54 (0.35)		-0.19 (0.13)		-0.68 (0.16)	***
Salary increase (10%)	0.30 (0.02)	***	0.19 (0.03)	***	0.97 (0.18)	***	0.74 (0.17)	***	0.44 (0.03)	***	0.22 (0.03)	***	0.50 (0.03)	***	0.25 (0.02)	***
Staff & resources available	1.65 (0.15)	***	1.32 (0.16)	***	5.06 (0.94)	***	2.69 (0.48)	***	1.72 (0.16)	***	1.01 (0.16)	***	2.57 (0.15)	***	1.33 (0.09)	***
Competent management	0.46 (0.09)	***	0.49 (0.20)	*	0.90 (0.22)	***	1.16 (0.38)	**	0.47 (0.09)	***	0.81 (0.19)	***	0.43 (0.06)	***	0.68 (0.11)	***
RWOPS: 8hrs/week after hrs ^a^	—		—		—		—		—		—		—		—	
RWOPS:16hrs/week after hrs	0.15 (0.12)		-0.70 (0.34)	*	0.06 (0.22)		-1.05 (0.42)	*	0.16 (0.12)		-0.17 (0.18)		-0.09 (0.13)		0.36 (0.30)	
RWOPS: 8hrs/week during work	0.03 (0.13)		-0.46 (0.71)		-0.51 (0.34)		0.32 (0.62)		-0.07 (0.14)		0.35 (0.31)		-0.24 (0.13)		0.16 (0.72)	
RWOPS prohibited	-1.39 (0.19)	***	-1.27 (0.17)	***	-1.42 (0.42)	**	-2.38 (0.59)	***	-1.75 (0.22)	***	1.18 (0.22)	***	-0.96 (0.14)	***	1.09 (0.13)	***
Doctorsx Part-time post													0.71 (0.19)	***		
Therapistsx Part-time post													0.57 (0.19)	**		
Doctorsx 8hrs overtime													0.74 (0.16)	***		
Therapistsx 8hrs overtime													0.50 (0.18)	**		
Doctorsx 16hrs overtime													1.01 (0.20)	***		
Therapistsx 16hrs overtime													0.65 (0.20)	**		
Doctorsx 10% Salary increase													-0.21 (0.03)	***		
Therapistsx 10% Salary increase													-0.06 (0.03)			
Doctorsx Staff & resources													-0.94 (0.16)	***		
Therapistsx Staff & resources													-0.84 (0.16)	***		
Doctorsx 16hrs RWOPS after hrs													0.29 (0.18)			
Therapistsx 16hrs RWOPS after hrs													0.25 (0.18)			
Doctorsx 8hrs RWOPS during work													0.28 (0.18)			
Therapistsx 8hrs RWOPS during work													0.22 (0.18)			
Doctorsx RWOPS prohibited													-0.32 (0.19)			
Therapistsx RWOPS prohibited													-0.75 (0.21)	***		
Opt-out	1.19 (0.41)	**	2.75 (0.26)	***	0.14 (0.39)		2.30 (0.31)	***	1.04 (0.51)	*	2.47 (0.39)	***	0.79 (0.30)	**	2.49 (0.32)	***
Tau	-0.01 (0.07)				0.95 (0.15)	***			-0.25 (0.70)	***			0.17 (0.14)			
*n*	*11 616*				*13 536*				*8 136*				*33 288*			
*LL*	*-2 866.90*				*-2 440.85*				*-1 773.42*				*-7 133.29*			
*AIC*	*5 775.81*				*4 923.70*				*3 588.83*				*14 340.59*			
*BIC*	*5 930.37*				*5 081.48*				*3 735.92*				*14 651.86*			
*p value*	*<0.001*				*<0.001*				*<0.001*				*<0.001*			

*p<0.05 **p<0.01 ***p<0.001, ^a^ Reference category, Therapists – rehabilitation therapists.

In the stratified analyses (models 3.1, 3.2, and 3.3), none of the groups preferred the more permissive RWOPS regimes (16 hours per week after hours or 8 hours per week during work hours) compared to the status quo of 8 hours per week after hours. However, there was a large and statistically significant aversion to jobs that prohibited RWOPS in all three groups. The interaction model (model 3.4) showed that this aversion was stronger among rehabilitation therapists and doctors compared to nurses, although only statistically significant for rehabilitation therapists.

As an alternative to restricting MJH, the DCE included several attributes to make public sector jobs more attractive. However, the option of moving to a part-time post with fewer hours in the primary job was not appealing, the coefficient was negative for all three groups although only statistically significant for nurses and rehabilitation therapists. The interaction terms for this attribute in the pooled model indicate that nurses were significantly less enthusiastic about a part-time post than both doctors and rehabilitation therapists (model 3.4). In terms of financial incentives, doctors and rehabilitation therapists strongly preferred jobs with additional overtime but nurses did not. All three professional groups derived significantly higher utility from jobs with higher salaries. The coefficient for a 10% salary increase was highest for nurses, but only the difference with doctors was statistically significant in the pooled model. Regarding non-financial incentives, a positive practice environment with available staff and resources was the most important attribute determining the job choices of the three groups. The coefficients for this attribute were significantly larger than those for a 10% salary increase. Although highly influential for all three groups in the separate models, the interaction terms in the pooled model indicate that the practice environment was significantly more valued by nurses than doctors and rehabilitation therapists (model 3.4). Competent management able to manage RWOPS was also a statistically significant determinant of job choices in all three professional groups (models 3.1, 3.2, and 3.3).

#### Marginal willingness to pay.

**Table 4** presents the basic DCE results by professional group in terms of marginal willingness to pay (mWTP). A negative mWTP indicates the amount of salary compensation respondents would require on average to accept an unattractive job attribute, whereas a positive mWTP represents the amount of salary that respondents would be prepared to forgo to keep a positive attribute. According to the DCE results, doctors would require a 45.7% salary increase to accept a job that prohibits MJH, whereas rehabilitation therapists would need 42.8%, and nurses 20.0%. At the other extreme, for a job with adequate resources and staffing which enabled them to provide quality care to patients, doctors, nurses, and rehabilitation therapists were willing to forgo 57.9%, 54.8%, and 38.9% of their salaries respectively. Similarly, for competent hospital management able to manage MJH, doctors, nurses, and rehabilitation therapists were ready to trade 13.7%, 7.7%, and 11.6% of their salaries, respectively. In terms of overtime opportunities, doctors equated a job with 16 hours of overtime to a 23.7% increase in salary, while it was 6.7% for rehabilitation therapists (**Table 4**).

**Table 4:** Marginal willingness to pay (mWTP), by professional group.

	Doctors		Nurses		Rehabilitation therapists	
	mWTP	95% CI	mWTP	95% CI	mWTP	95% CI
Part-time post	-8.2	[-17.9, 1.5]	-15.3	[-21.9, -8.7]	-9.5	[-16.3, -2.7]
8 hrs/week paid OT	21.2	[12.7, 29.8]	-3.3	[-8.1, 1.5]	6.6	[0.3, 12.9]
16 hrs/week paid OT	23.7	[13.1, 34.3]	-5.0	[-9.8, -0.2]	6.7	[0.0, 13.3]
Staff & resources available	57.9	[48.7, 67.1]	54.8	[49.1, 60.5]	38.9	[33.7, 44.1]
Competent management	13.7	[7.7, 19.6]	7.7	[3.9, 11.4]	11.6	[6.9, 16.4]
RWOPS: 16 hrs/week, after work	7.0	[-1.6, 15.6]	-3.1	[-8.5, 2.3]	3.6	[-2.0, 9.2]
RWOPS: 8 hrs/week, during work	3.0	[-5.7, 11.7]	-4.1	[-9.8, 1.5]	-1.8	[-8.0, 4.4]
RWOPS prohibited	-45.7	[-56.3, -35.0]	-20.0	[-26.0, -13.9]	-42.8	[-51.0, -34.6]

mWTP expressed as % increase in salary, results from G-MNL model in WTP space, OT: overtime.

#### Predicted job uptake.

**Table 5** uses the G-MNL DCE results to model the impact of different combinations of attributes on job uptake by the different professional groups. This allows us to evaluate the effectiveness of different compensatory interventions which could be used to offset the impact of an MJH ban. The selected baseline job for comparison was a full-time post without overtime and no salary increase, with an inadequate clinical practice environment and sub-optimal hospital management but allowing 8 hours of RWOPS after normal hours. The model estimated the uptake of such a job to be 22.0% for doctors, 28.8% for nurses and 22.7% for rehabilitation therapists.

**Table 5:** Modelling the impact of different financial and non-financial incentives to counteract a ban on MJH, by professional group.

Attributes	Baseline	Ban MJH	MJH Ban + Financial Incentives					MJH Ban + Non-Financial Incentives		
		*Scenario 1*	*Scenario 2*	*Scenario 3*	*Scenario 4*	*Scenario 5*	*Scenario 6*	*Scenario 7*	*Scenario 8*	*Scenario 9*
Conditions for RWOPS (MJH)	8hrs/week after hrs	Prohibited	Prohibited	Prohibited	Prohibited	Prohibited	Prohibited	Prohibited	Prohibited	Prohibited
Employment contract	F/T post, no paid OT	F/T post, no paid OT	F/T post, no paid OT	F/T post, no paid OT	F/T post, no paid OT	**8 hr** **OT/week**	**16 hrs OT/week**	F/T post, no paid OT	F/T post, no paid OT	F/T post, no paid OT
Salary increase	0%	0%	**25%**	**50%**	**75%**	0%	0%	0%	0%	0%
Clinical environment	Inadequate	Inadequate	Inadequate	Inadequate	Inadequate	Inadequate	Inadequate	**Adequate**	Inadequate	**Adequate**
Hospital management	Sub-optimal	Sub-optimal	Sub-optimal	Sub-optimal	Sub-optimal	Sub-optimal	Sub-optimal	Sub-optimal	**Competent**	**Competent**
Professional Group	**Uptake (%)**	**Change in Uptake from Baseline Scenario (Percentage points)**								
Doctors	22.0*%*	-11.0*%*	-4.1*%*	+5.2*%*	+15.1*%*	-5.5*%*	-2.8*%*	+8.0*%*	-6.7*%*	+13.7*%*
Nurses	28.8*%*	-6.8*%*	+15.6*%*	+32.1*%*	+41.2*%*	-6.1*%*	-6.7*%*	+35.3*%*	+1.6*%*	+40.5*%*
Rehab therapists	22.7*%*	-14.0*%*	-4.3*%*	+9.9*%*	+24.6*%*	-11.1*%*	-10.4*%*	+3.9*%*	-9.5*%*	+10.4*%*

Results from the G-MNL model stratified by professional group.

OT: overtime, Hrs: hours, MJH: multiple job holding.

If MJH were prohibited this would decrease job uptake by 11.0, 6.8 and 14.0 percentage points for doctors, nurses and rehabilitation therapists, respectively (Scenario 1). The remaining scenarios evaluate the financial and non-financial incentives required to counteract the impact of a ban on MJH. In terms of financial incentives, a salary increase of at least 50% (Scenario 3) would be required before the decrease in job uptake is reversed for all three groups. Allowing overtime in the primary job would moderate the impact of a ban but not compensate completely in any of the groups (Scenarios 4 and 5). The model indicates that non-financial incentives could persuade health professionals to stay in public posts that banned MJH. A positive practice environment that supports quality care would counteract the impact of a ban for doctors, nurses and rehabilitation therapists with increased uptake of 8.0, 35.3 and 3.9 percentage points above baseline respectively (Scenario 7). Competent hospital management would not be sufficient alone (Scenario 8) but would make an effective package when combined with an improved practice environment for doctors, nurses and rehabilitation therapists improving uptake by 13.7, 40.5 and 10.4 percentage points respectively (Scenario 9).

### DCE results by multiple job holding status

**Table 6** compares the G-MNL DCE results for health professionals who did not engage in MJH in the previous 12 months with those who did, in separate models for each group (models 6.1 and 6.2) as well as a pooled model including interactions between MJH practice and the DCE attributes (model 6.3). The significance and relative importance of the DCE attributes were comparable to the results in **Table 3** and similar between the MJH and non-MJH professionals. However, professionals who engaged in MJH were more strongly opposed to jobs which prohibited RWOPS than those who did not engage in MJH, which was confirmed to be statistically significant in model 6.3. Interestingly, the non-MJH professionals placed a significantly higher value on a 10% salary increase, a better clinical practice environment and more competent management than those currently engaged in MJH (model 6.3)

**Table 6:** DCE G-MNL results for professionals engaged and not engaged in MJH.

	*Model 6.1*				*Model 6.2*				*Model 6.3*			
	Non-MJH				MJH				Pooled + Interactions			
	Mean (SE)		SD (SE)		Mean (SE)		SD (SE)		Mean (SE)		SD (SE)	
Full-time post, no paid overtime ^a^	—		—		—		—		—		—	
Part-time post, no paid overtime	-0.51 (0.13)	***	0.66 (0.27)	*	-0.27 (0.17)		-0.75 (0.20)	***	-0.38 (0.08)	***	-0.51 (0.15)	**
Full-time post, 8hrs paid overtime	0.40 (0.11)	***	0.08 (0.08)		0.44 (0.17)	**	-0.19 (0.33)		0.35 (0.07)	***	0.17 (0.15)	
Full-time post, 16hrs paid overtime	0.41 (0.14)	**	0.75 (0.24)	**	0.49 (0.19)	*	-0.73 (0.27)	**	0.37 (0.09)	***	-0.72 (0.14)	***
Salary increase (10%)	0.52 (0.08)	***	0.33 (0.05)	***	0.35 (0.03)	***	0.22 (0.03)	***	0.43 (0.02)	***	0.27 (0.02)	***
Staff & resources available	2.57 (0.35)	***	1.65 (0.26)	***	1.58 (0.20)	***	1.23 (0.20)	***	2.16 (0.11)	***	1.35 (0.09)	***
Competent management	0.61 (0.12)	***	0.59 (0.20)	**	0.32 (0.12)	**	0.70 (0.22)	**	0.51 (0.06)	***	0.57 (0.122)	***
RWOPS: 8hrs/week after hrs ^a^	—		—		—		—		—		—	
RWOPS:16hrs/week after hrs	0.09 (0.10)		-0.46 (0.26)		0.17 (0.16)		0.82 (0.26)	**	0.10 (0.08)		0.37 (0.24)	
RWOPS: 8hrs/week during work	-0.20 (0.14)		0.12 (0.19)		0.02 (0.15)		0.20 (0.31)		-0.09 (0.09)		-0.09 (0.25)	
RWOPS prohibited	-1.21 (0.22)	***	1.31 (0.27)	***	-2.02 (0.26)	***	-1.28 (0.17)	***	-1.00 (0.11)	***	1.06 (0.12)	***
MJH x 10% Salary increase									-0.10 (0.03)	**		
MJH x Staff & resources									-0.72 (0.15)	***		
MJH x Competent management									-0.27 (0.11)	*		
MJH x 16 hrs RWOPS after hrs									0.06 (0.16)			
MJH x 8 hrs RWOPS during work									0.08 (0.16)			
MJH x RWOPS prohibited									-0.88 (0.19)	***		
Opt-out	0.43 (0.29)		2.38 (0.27)	***	1.45 (0.46)	**	2.77 (0.43)	***	0.65 (0.25)	*	2.59 (0.23)	***
Tau	-0.56 (0.15)	***			0.09 (0.10)				0.20 (0.12)			
*n*	*25 632*						*7 656*		*33 288*			
*LL*	*-5 213.26*						*-1 973.61*		*-7 210.55*			
*AIC*	*10 468.52*						*3 989.22*		*14 445.11*			
*BIC*	*10 639.70*						*4 135.03*		*14 702.26*			
*p value*	*<0.001*						*<0.001*		*<0.001*			

*p<0.05 **p<0.01 ***p<0.001, ^a^ Reference category, MJH: engaged in multiple job holding, Non-MJH: not engaged in multiple job holding

### DCE results for different socio-demographic groups

We ran a pooled G-MNL model with socio-demographic interactions to evaluate their impact on attribute preferences (Supplemental File 3). There were no significant differences between male and female doctors, nurses, and rehabilitation therapists in avoiding jobs that prohibited MJH. Married nurses did not differ from single nurses in their preference for jobs that prohibited MJH. Furthermore, generalist doctors valued salary increases more than specialists, whereas nurses from Gauteng province (GP) valued salary increases more than those in Mpumalanga province (MP). All sub-groups preferred a supportive clinical environment, but this preference was significantly stronger among female doctors, female rehabilitation therapists, and nurses from GP.

## Discussion

We presented the results of a novel DCE evaluating the preferences of South African public sector medical doctors, professional nurses and rehabilitation therapists for different MJH regulatory interventions. We found that all three groups were significantly opposed to a ban on MJH (**Table 3**). They would refuse jobs that did not permit MJH or require a substantial increase in salary to accept them. Interestingly, none of the groups were clearly in favour of doubling the hours of permitted MJH or allowing MJH during working hours.

All three professional groups valued salary increases highly, and improved basic remuneration would significantly increase the uptake of public sector jobs. Doctors and rehabilitation therapists were prepared to trade higher salaries for increased overtime allowances, but nurses were not. None of the groups were in favour of part-time posts, even though it might be a more accurate representation of the current split between primary and secondary jobs and would allow more official time in their second private jobs. Improving the clinical practice environment, with adequate staffing and resources to ensure quality patient care, would be a powerful non-financial incentive to increase public sector retention. All three professional groups were prepared to forgo a large proportion of their salaries for such improvements. More competent hospital management was slightly less important but still a significant determinant of job choices.

There is some support from the DCE that a ban on MJH could be offset by improvements in other job characteristics. The respondents accepted posts that did not permit MJH if there was adequate compensation. A significantly increased salary or a well-resourced clinical environment were sufficient alone to overcome the health professional’s aversion to an MJH ban, but additional overtime or better hospital management would need to be combined with other financial or non-financial incentives. The ranking of attributes was similar between the three professional groups but there were some statistically significant differences in the magnitude of their preferences. The coefficients for doctors and rehabilitation therapists were mostly comparable to each other. However, the nurses were significantly less enthusiastic about part-time posts and overtime and valued the salary increase and clinical practice environment more highly, than the other two groups. The preferences of health professionals who had engaged in MJH in the previous year were also qualitatively similar to those that had not (**Table 6**). However, MJH professionals were significantly more opposed to a MJH ban and valued the salary increase and clinical practice environment less than the non-MJH professionals.

This research makes an important contribution to the small literature on the opinions of health professionals about MJH regulation. It is the first study to use a DCE to directly quantify the trade-offs between different MJH policy interventions in the health sector. It is also innovative in comparing doctors, nurses and rehabilitation therapists, and achieved a good response rate of 83.9% which limits selection bias. Other methodological strengths were that we used a rigorous G-MNL model for the DCE analysis and statistically tested differences in preferences between groups through interaction models.

The study also has several limitations. DCEs are often criticised for measuring stated rather than revealed preferences, although there is some recent evidence of their external validity in predicting real choices [56]. Due to both statistical and cognitive constraints, DCEs are always limited in the number of attributes or levels that can be included. We focused on policy interventions relevant to the SA context, as identified by key informants and health professionals in the qualitative interviews. The study was based in only two provinces and may not be generalisable to the entire country, although Gauteng and Mpumalanga were chosen because they are fairly representative of urban and rural provinces respectively.

The likely effectiveness of different MJH regulations will vary from one context to another. Many authors have proposed that MJH interventions in low- and middle-income countries (LMICs) will need to be different to those in high-income countries (HICs) [5,6,17,18,21,57]. For example, Garcia-Prado & Gonzalez [9] suggest that *reward-oriented* MJH policies are more appropriate in HICs, while *restrictive* policies are recommended for LMICs.

Health policymakers and managers frustrated with the absenteeism of public sector employees engaged in MJH, may be supportive of an outright ban of MJH [58], and this has been tried in a few countries [18]. The theoretical models provide inconclusive advice. Brekke and Sørgard [23] found that an MJH ban may be efficient under certain conditions, while Gonzalez & Macho-Stadler [21] conclude that banning MJH is never desirable, even if a ban could be enforced. However, the empirical country case studies suggest that an MJH ban has limited impact because it is difficult to enforce, particularly in LMICs but even in HICs with stronger regulatory capacity [18]. The risk is that a complete MJH ban might lead to an exodus of skilled health professionals from the public sector to the private sector, undermining the objective of such an intervention which would be to increase access and quality of care for public patients. The respondents in our DCE were opposed to an MJH ban, and the model predicts a significant impact on job uptake. However, the size of the shift was perhaps not as large as might have been expected from the threats of professional groups opposed to a ban [59]. There may be sufficient advantages for staying in a public post in South Africa (SA) even if MJH is banned, or it could be that health professionals are not completely convinced that a ban would be enforced. The fact that compliance with current RWOPS regulations in SA is low with seemingly few consequences [35,58], may support the latter reasoning.

Pay differentials between the public and private health sectors are a key driver of MJH and increased public sector salaries might make MJH less necessary for health professionals. Unsurprisingly, increased salary levels strongly influenced job choices in our DCE. We also found that doctors, nurses and rehabilitation therapists would require salary increases of 45.7%, 20.0% and 42.8% respectively, to accept a post that did not allow MJH. In Gruen *et al*’s [60] survey of MJH doctors in Bangladesh, 100% of primary care doctors and 54% of doctors in secondary and tertiary care said they would give up MJH completely if they were paid higher government salaries. However, the amount of the increase required was not specified. Similarly, in Do and Do’s [61] survey of MJH doctors in Vietnam, 65% of doctors said they would give up their private practices under specific conditions. Approximately 29% of those willing to leave private practice desired a higher basic pay, and the proposed increase was 2.7 times their current pay. Unfortunately, fiscal constraints mean that increasing public sector remuneration is seldom affordable, particularly in LMICs where pay differentials between the public and private sectors are large and government revenue is low [9,18]. Even in HICs that have been able to increase public sector salaries, the evidence is mixed. Sæther [Unpublished work] used a discrete choice model to analyse the revealed preferences of Norwegian doctors and found that a 10% increase in public sector salary levels resulted in a statistically significant but small shift in time allocation away from private practices towards public sector work. However, Mossialos *et al* [24] report that a raise in public doctor salaries had little impact on MJH in Greece. SA has also already experimented with similar reforms. The so-called Occupational Specific Dispensation (OSD) policy was an increase in public sector salaries, implemented for different categories of health professionals from 2007 to 2009, to improve public sector retention. Unfortunately, the policy was not formally evaluated, and the effects on retention and MJH are equivocal [62,63]. Furthermore, the national economic and fiscal climate has been much less favourable in recent years [64], which makes any significant increase in government salaries extremely unlikely in the near future.

Although economic motivations are generally the most important, it is widely accepted that other non-financial considerations play a role in the decision of health professionals to engage in MJH [5]. A second job in the private sector may provide job complementarity, diversity, autonomy, the learning of new skills or career development [5,7,8,43]. Our previous descriptive analysis, confirms those motivations for MJH in this sample of health professionals [46]. Institutional factors are also relevant [5,9]. For example, Hoogland *et al* [6] found that poor working conditions, inadequate facilities and shortages of drugs and equipment in the public sector, were noted as reasons for MJH by doctors in approximately one-third of the 157 countries included in their review, with similar levels of complaints from both LMICs and HICs. However, despite this recognition, non-financial interventions have received much less attention in the literature on possible MJH policies [17,18,21]. Non-financial attributes were as important as financial attributes in this DCE. Improved public sector working conditions and facility management would be effective interventions in increasing public sector retention, even if MJH was banned. However, it must also be recognised that these are not simple problems to address in SA. Declining public health budgets and the deterioration of public health facilities and services have been noted for years [28,29], as has the poor monitoring and regulation of MJH by public health managers [35,65,66].

Lastly, our conclusions about the preferences of MJH and non-MJH professionals were comparable to those of Pestana *et al* [44]. They also found that the two groups had similar preferences and that professionals working exclusively in the public sector were significantly more concerned about the clinical practice environment. This would support the arguments for targeting non-financial interventions to increase public sector retention and decrease MJH.

## Conclusions

Our DCE study provides new insights into the preferences of different groups of health professionals for different MJH regulatory interventions and confirms the importance of non-financial considerations in MJH decision-making. Such information is useful in the design and tailoring of MJH regulation, particularly in LMICs. However, significantly improved government finances, management and regulatory capacity will be required for the successful implementation of any MJH interventions.

## Supporting information

S1 File Choice task instructions. (TIF)

S2 File Comparison of the c-logit, MIXL and uncorrelated G-MNL models. (DOCX)

S3 File Interaction model of socio-demographic factors. (DOCX)
