# Supplementary figures and images for: Preferences of public sector medical doctors, professional nurses and rehabilitation therapists for multiple job holding regulation: A discrete choice experiment
Source: PLoS One. 2025 Apr 15;20(4):e0320854. doi: 10.1371/journal.pone.0320854 (PMC11999164; doi:10.1371/journal.pone.0320854)

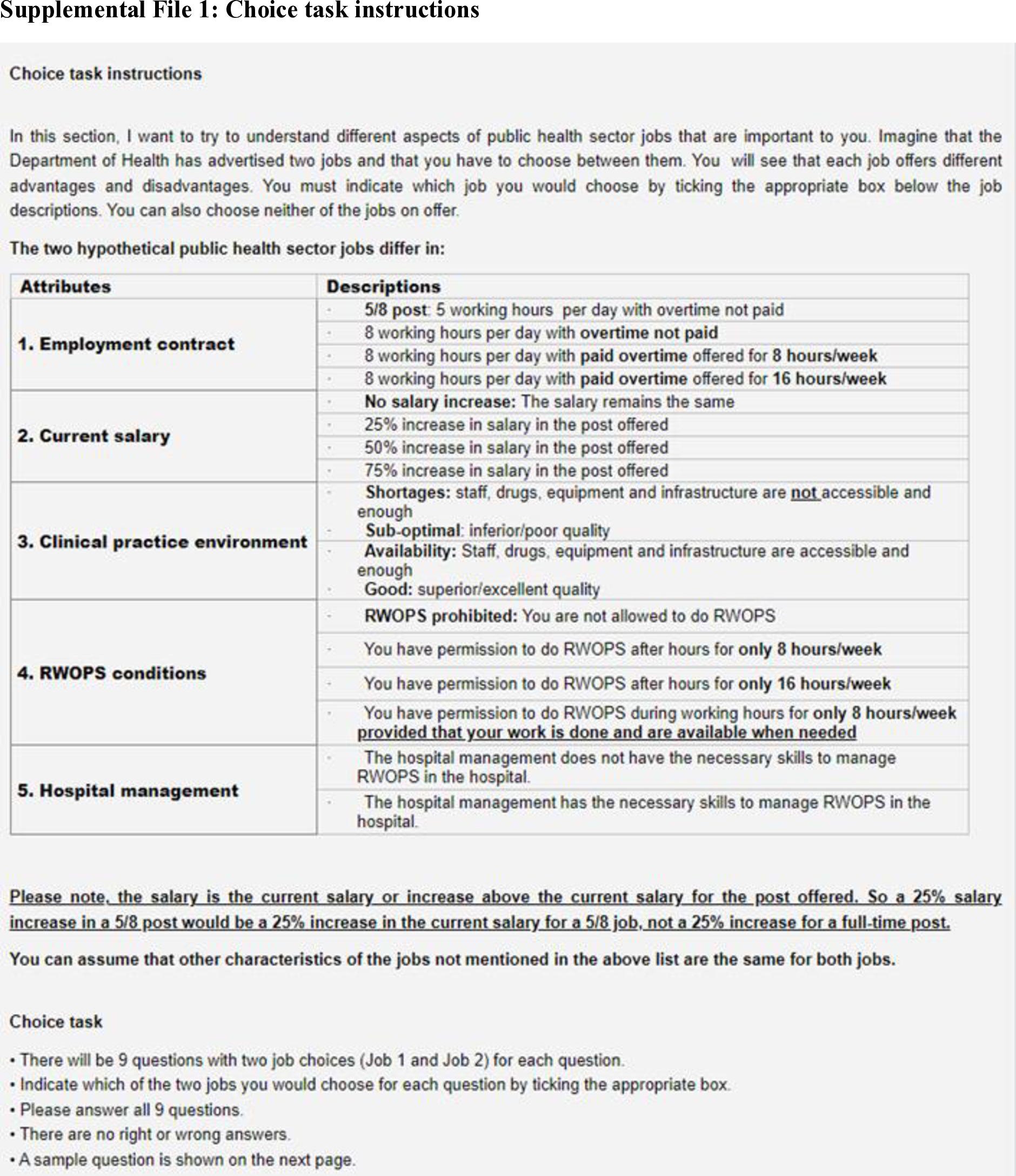

Supplement: S1 File — (TIF) [file pone.0320854.s001.tif]
