# Supplementary material for: Preferences of public sector medical doctors, professional nurses and rehabilitation therapists for multiple job holding regulation: A discrete choice experiment
Source: PLoS One. 2025 Apr 15;20(4):e0320854. doi: 10.1371/journal.pone.0320854 (PMC11999164; doi:10.1371/journal.pone.0320854)
Supplement: S2 File — (DOCX) [file pone.0320854.s002.docx]

**Supplemental File 2: Comparison of the conditional logit, MIXL and uncorrelated G-MNL model**

| Medical doctors | Clogit | |  | MXL | | | |  | G-MNL | | | |
| --- | --- | --- | --- | --- | --- | --- | --- | --- | --- | --- | --- | --- |
|  | **Mean (SE)** |  |  | **Mean (SE)** |  | **SD (SE)** |  |  | **Mean (SE)** |  | **SD (SE)** |  |
| Full-time post, no paid overtime | — |  |  | — |  | — |  |  | — |  | — |  |
| Part-time post, no paid overtime | -0.15 (0.09) |  |  | -0.16 (0.13) |  | -0.56 (0.25) | * |  | -0.20 (0.14) |  | 0.47 (0.38) |  |
| Full-time post, 8hrs paid overtime | 0.39 (0.09) | *** |  | 0.61 (0.12) | *** | -0.02 (0.19) |  |  | 0.63 (0.13) | *** | 0.17 (0.25) |  |
| Full-time post, 16hrs paid overtime | 0.44 (0.09) | *** |  | 0.75 (0.16) | *** | 0.84 (0.25) | ** |  | 0.78 (0.17) | *** | 0.90 (0.19) | *** |
| Salary increase (10%) | 0.19 (0.01) | *** |  | 0.28 (0.02) | *** | 0.16 (0.03) | *** |  | 0.30 (0.02) | *** | 0.19 (0.03) | *** |
| Staff & resources available | 1.08 (0.06) | *** |  | 1.60 (0.14) | *** | 1.16 (0.15) | *** |  | 1.65 (0.15) | *** | 1.32 (0.16) | *** |
| Competent management | 0.25 (0.06) | *** |  | 0.45 (0.09) | *** | 0.32 (0.21) |  |  | 0.46 (0.09) | *** | 0.49 (0.20) | * |
| RWOPS: 8hrs/week after hrs | - |  |  | - |  | - |  |  | — |  | — |  |
| RWOPS: 16hrs/week after hrs | 0.15 (0.08) |  |  | 0.14 (0.12) |  | -0.79 (1.30) | ** |  | 0.15 (0.12) |  | -0.70 (0.34) | * |
| RWOPS: 8hrs/week during work | 0.06 (0.09) |  |  | 0.04 (0.13) |  | 0.49 (0.28) |  |  | 0.03 (0.13) |  | -0.46 (0.71) |  |
| RWOPS prohibited | -0.85 (0.10) | *** |  | -1.32 (0.17) | *** | 1.22 (0.25) | *** |  | -1.39 (0.19) | *** | -1.27 (0.17) | *** |
| Opt-out | 1.40 (0.16) | *** |  | 1.01 (0.43) | * | 2.59 (0.30) | *** |  | 1.19 (0.41) | ** | 2.75 (0.26) | *** |
| Tau |  |  |  |  |  |  |  |  | -0.01 (0.07) |  |  |  |
| N | *11 616* |  |  | *11 616* |  |  |  |  | *11 616* |  |  |  |
| LL | *-3 357.68* |  |  | *-2 873.61* |  |  |  |  | *-2 866.90* |  |  |  |
| AIC | *6 735.36* |  |  | *5 787.21* |  |  |  |  | *5 775.81* |  |  |  |
| BIC | *6 808.96* |  |  | *5 934.42* |  |  |  |  | *5 930.37* |  |  |  |
| p value | *<0.001* | ***** |  | *<0.001* | *** |  |  |  | *<0.001* |  |  |  |

*p<0.05 **p<0.01 ***p<0.001, ^a^ Reference category

| Professional nurses | Clogit | |  | MXL | | | |  | G-MNL | | | |
| --- | --- | --- | --- | --- | --- | --- | --- | --- | --- | --- | --- | --- |
|  | **Mean (SE)** |  |  | **Mean (SE)** |  | **SD (SE)** |  |  | **Mean (SE)** |  | **SD (SE)** |  |
| Full-time post, no paid overtime | — |  |  | — |  | — |  |  | — |  | — |  |
| Part-time post, no paid overtime | -0.50 (0.09) | *** |  | -0.64 (0.13) | *** | 0.30 (0.24) | * |  | -1.30 (0.33) | *** | -1.11 (0.57) |  |
| Full-time post, 8hrs paid overtime | -0.13 (0.09) |  |  | -0.04 (0.10) |  | 0.03 (0.11) |  |  | 0.08 (0.26) |  | -0.36 (0.51) |  |
| Full-time post, 16hrs paid overtime | -0.15 (0.09) |  |  | -0.13 (0.13) |  | 0.26 (0.33) |  |  | -0.19 (0.25) |  | -1.36 (0.49) | ** |
| Salary increase (10%) | 0.29 (0.01) | *** |  | 0.46 (0.03) | *** | 0.31 (0.04) | *** |  | 0.97 (0.18) | *** | 0.74 (0.17) | *** |
| Staff & resources available | 1.58 (0.06) | *** |  | 2.38 (0.18) | *** | 1.46 (0.15) | *** |  | 5.06 (0.94) | *** | 2.69 (0.48) | *** |
| Competent management | 0.20 (0.05) | *** |  | 0.40 (0.09) | *** | 0.63 (0.20) | ** |  | 0.90 (0.22) | *** | 1.16 (0.38) | ** |
| RWOPS: 8hrs/week after hrs | — |  |  | — |  | — |  |  | — |  | — |  |
| RWOPS: 16hrs/week after hrs | -0.08 (0.08) |  |  | -0.00 (0.13) |  | 0.25 (0.65) |  |  | 0.06 (0.22) |  | -1.05 (0.42) | * |
| RWOPS: 8hrs/week during work | -0.06 (0.09) |  |  | -0.11 (0.12) |  | -0.00 (0.17) |  |  | -0.51 (0.34) |  | 0.32 (0.62) |  |
| RWOPS prohibited | -0.50 (0.10) | *** |  | -0.68 (0.14) | *** | -0.74 (0.26) | ** |  | -1.42 (0.42) | ** | -2.38 (0.59) | *** |
| Opt-out | 0.71 (0.16) | *** |  | -0.02 (0.48) |  | 2.40 (0.38) | *** |  | 0.14 (0.39) |  | 2.30 (0.31) | *** |
| Tau |  |  |  |  |  |  |  |  | 0.95 (0.15) | *** |  |  |
| N | *13 536* |  |  | *13 536* |  |  |  |  | *13 536* |  |  |  |
| LL | *-2 837.72* |  |  | *-2 465.56* |  |  |  |  | *-2 440.85* |  |  |  |
| AIC | *5 695.44* |  |  | *4 971.12* |  |  |  |  | *4 923.70* |  |  |  |
| BIC | *5 770.57* |  |  | *5 121.38* |  |  |  |  | *5 081.48* |  |  |  |
| p value | *<0.001* | *** |  | *<0.001* | *** |  |  |  | *<0.001* |  |  |  |

*p<0.05 **p<0.01 ***p<0.001, ^a^ Reference category

| Rehabilitation therapists (RTs) | Clogit | |  | MXL | | |  |  | G-MNL | | | |
| --- | --- | --- | --- | --- | --- | --- | --- | --- | --- | --- | --- | --- |
|  | **Mean (SE)** |  |  | **Mean (SE)** |  | **SD (SE)** |  |  | **Mean (SE)** |  | **SD (SE)** |  |
| Full-time post, no paid overtime | — |  |  | — |  | — |  |  | — |  | — |  |
| Part-time post, no paid overtime | -0.20 (0.09) | * |  | -0.32 (0.14) | * | 0.73 (0.38) |  |  | -0.32 (0.15) | * | 0.80 (0.28) | ** |
| Full-time post, 8hrs paid overtime | 0.21 (0.09) | * |  | 0.35 (0.13) | ** | -0.18 (0.36) |  |  | 0.40 (0.13) | ** | -0.27 (0.32) |  |
| Full-time post, 16hrs paid overtime | 0.23 (0.09) | * |  | 0.40 (0.14) | ** | -0.57 (0.24) | * |  | 0.43 (0.15) | ** | -0.54 (0.35) |  |
| Salary increase (10%) | 0.27 (0.01) | *** |  | 0.41 (0.03) | *** | 0.22 (0.04) | *** |  | 0.44 (0.03) | *** | 0.22 (0.03) | *** |
| Staff & resources available | 1.13 (0.07) | *** |  | 1.65 (0.16) | *** | 0.92 (0.16) | *** |  | 1.72 (0.16) | *** | 1.01 (0.16) | *** |
| Competent management | 0.26 (0.06) | *** |  | 0.41 (0.10) | *** | 0.84 (0.18) | *** |  | 0.47 (0.09) | *** | 0.81 (0.19) | *** |
| RWOPS: 8hrs/week after hrs | — |  |  | — |  | — |  |  | — |  | — |  |
| RWOPS: 16hrs/week after hrs | 0.12 (0.08) |  |  | 0.15 (0.11) |  | -0.30 (0.16) |  |  | 0.16 (0.12) |  | -0.17 (0.18) |  |
| RWOPS: 8hrs/week during work | 0.01 (0.10) |  |  | -0.08 (0.13) |  | 0.41 (0.21) |  |  | -0.07 (0.14) |  | 0.35 (0.31) |  |
| RWOPS prohibited | -1.07 (0.11) | *** |  | -1.63 (0.20) | *** | 1.06 (0.19) | *** |  | -1.75 (0.22) | *** | 1.18 (0.22) | *** |
| Opt-out | 1.29 (0.18) | *** |  | 1.12 (0.63) |  | 2.15 (0.35) | *** |  | 1.04 (0.51) | * | 2.47 (0.39) | *** |
| Tau |  |  |  |  |  |  |  |  | -0.25 (0.70) | *** |  |  |
| N | *8 136* |  |  | *8 136* |  |  |  |  | *8 136* |  |  |  |
| LL | *-1 999.32* |  |  | *-1 776.11* |  |  |  |  | *-1 773.42* |  |  |  |
| AIC | *4 018.64* |  |  | *3 592.23* |  |  |  |  | *3 588.83* |  |  |  |
| BIC | *4 088.68* |  |  | *3 732.31* |  |  |  |  | *3 735.92* |  |  |  |
| p value | *<0.001* | *** |  | *<0.001* | *** |  |  |  | *<0.001* |  |  |  |

*p<0.05 **p<0.01 ***p<0.001, ^a^ Reference category
