# Supplementary material for: Preferences of public sector medical doctors, professional nurses and rehabilitation therapists for multiple job holding regulation: A discrete choice experiment
Source: PLoS One. 2025 Apr 15;20(4):e0320854. doi: 10.1371/journal.pone.0320854 (PMC11999164; doi:10.1371/journal.pone.0320854)
Supplement: S3 File — (DOCX) [file pone.0320854.s003.docx]

**Supplemental File 3: Pooled model with interactions with socio-demographic factors**

**Medical doctors**

| Attributes | Mean (SE) | | SD (SE) | |
| --- | --- | --- | --- | --- |
| Full-time post ^a^ | — |  | — |  |
| Part-time post | -0.16 (0.14) |  | 0.53 (0.19) | *** |
| 8hrs paid overtime | 0.66 (0.13) | *** | 0.24 (0.19) |  |
| 16hrs paid overtime | 0.84 (0.17) | *** | 0.98 (0.24) | *** |
| Salary increase (10%) | 0.36 (0.04) | *** | -0.18 (0.03) | *** |
| Staff & resources available | 2.57 (0.31) | *** | 1.21 (0.16) | *** |
| Competent management | 0.47 (0.10) | *** | 0.43 (0.19) | * |
| RWOPS: 8hrs/week after hours ^a^ | — |  | — |  |
| RWOPS:16hrs/week after hrs | -0.19 (0.19) |  | 0.81 (0.18) | *** |
| RWOPS: 8hrs/week during work | 0.03 (0.13) |  | 0.63 (0.33) |  |
| RWOPS prohibited | -1.33 (0.26) | *** | -1.28 (0.20) | *** |
|  |  |  |  |  |
| Male x Staff & resources | -1.04 (0.21) | *** |  |  |
| Dependents x Staff & resources | -0.34 (0.27) |  |  |  |
| Married x 16 hrs/week after hrs | 0.61 (0.23) | ** |  |  |
| Specialists x 10% Salary increase | -0.14 (0.04) | ** |  |  |
| Male x RWOPS prohibited | -0.36 (0.24) |  |  |  |
| GP x RWOPS prohibited | 0.14 (0.23) |  |  |  |
|  |  |  |  |  |
| Opt-out | 1.31 (0.42) | ** | 2.76 (0.26) | *** |
| Tau | 0.10 (0.10) |  |  |  |
| N | 11 568 |  |  |  |
| LL | -2 790.20 |  |  |  |
| AIC | 5 634.40 |  |  |  |
| BIC | 5 833.01 |  |  |  |
| p-value | <0.001 |  |  |  |

*p<0.05 **p<0.01 ***p<0.001, ^a^ Reference category

MJH – multiple job holding, GP – Gauteng province

**Professional nurses**

| Attributes | Mean (SE) | | SD (SE) | |
| --- | --- | --- | --- | --- |
| Full-time post ^a^ | — |  | — |  |
| Part-time post | -0.87 (0.18) | *** | 0.57 (0.32) |  |
| 8hrs paid overtime | 0.03 (0.16) |  | 0.14 (0.19) |  |
| 16hrs paid overtime | -0.06 (0.17) |  | 0.27 (0.36) |  |
| Salary increase (10%) | 0.58 (0.08) | *** | 0.46 (0.10) | *** |
| Staff & resources available | 2.74 (0.38) | *** | 1.88 (0.24) | *** |
| Competent management | 0.56 (0.14) | *** | 0.94 (0.24) | *** |
| RWOPS: 8hrs/week after hours ^a^ | — |  | — |  |
| RWOPS:16hrs/week after hrs | -0.00 (0.18) |  | 0.30 (0.42) |  |
| RWOPS: 8hrs/week during work | 1.92 (1.13) |  | -0.55 (0.35) |  |
| RWOPS prohibited | -1.89 (0.22) | *** | -1.14 (0.30) |  |
|  |  |  |  |  |
| Male x 16hrs paid overtime | -0.88 (0.53) |  |  |  |
| Specialised x 10% Salary increase | -0.12 (0.07) |  |  |  |
| GP x 10% Salary increase | 0.13 (0.06) | * |  |  |
| Male x Staff & resources | -0.94 (0.54) |  |  |  |
| GP x Staff & resources | 0.80 (0.24) | ** |  |  |
| Dependents x 8hrs RWOPS during work | -2.04 (1.15) |  |  |  |
| Specialised x 8hrs RWOPS during work | -0.63 (0.27) | * |  |  |
| Male x RWOPS prohibited | 0.68 (0.51) |  |  |  |
| Married x RWOPS prohibited | -0.27 (0.31) |  |  |  |
|  |  |  |  |  |
| Opt-out | -0.04 (0.42) |  | 2.60 (0.43) | *** |
| Tau | 0.59 (0.12) | *** |  |  |
| N | 13 512 |  |  |  |
| LL | -2 414.08 |  |  |  |
| AIC | 4 888.16 |  |  |  |
| BIC | 5 113.50 |  |  |  |
| p-value | <0.001 |  |  |  |

*p<0.05 **p<0.01 ***p<0.001, ^a^ Reference category

MJH – multiple job holding, GP – Gauteng province

**Rehabilitation therapists**

| Attributes | Mean (SE) | | SD (SE) | |
| --- | --- | --- | --- | --- |
| Full-time post ^a^ | — |  | — |  |
| Part-time post | -0.51 (0.17) | ** | 0.77 (0.29) | ** |
| 8hrs paid overtime | 0.40 (0.13) | ** | -0.28 (0.37) |  |
| 16hrs paid overtime | 0.44 (0.14) | ** | 0.60 (0.23) | ** |
| Salary increase (10%) | 0.46 (0.04) | *** | 0.22 (0.03) | *** |
| Staff & resources available | 1.79 (0.15) | *** | 0.99 (0.14) | *** |
| Competent management | 0.47 (0.11) | *** | 0.79 (0.15) | *** |
| RWOPS: 8hrs/week after hours ^a^ | — |  | — |  |
| RWOPS:16hrs/week after hrs | 0.17 (0.12) |  | -0.35 (0.16) | * |
| RWOPS: 8hrs/week during work | -0.07 (0.13) |  | 0.29 (0.38) |  |
| RWOPS prohibited | -1.67 (0.21) | *** | 1.12 (0.16) | *** |
|  |  |  |  |  |
| Male x part-time post | 0.50 (0.23) | * |  |  |
| Male x Staff & resources | -0.52 (0.25) | * |  |  |
| Married x Salary increase | -0.07 (0.04) |  |  |  |
| Male x RWOPS prohibited | -0.34 (0.45) |  |  |  |
|  |  |  |  |  |
| Opt-out | 0.73 (0.64) |  | 2.68 (0.57) | *** |
| Tau | 0.21 (0.06) | ** |  |  |
| N | 8 016 |  |  |  |
| LL | -1 726.68 |  |  |  |
| AIC | 3 503.36 |  |  |  |
| BIC | 3 678.09 |  |  |  |
| p-value | <0.001 |  |  |  |

*p<0.05 **p<0.01 ***p<0.001, ^a^ Reference category

MJH – multiple job holding
